# The Physiological Cost of the Boat-Race

**Published:** 1896-04-11

**Authors:** 


### The Physiological Cost of the Boat-Race.

We publish the substance of the following letters which have appeared in the *Standard*, and have dealt with the points raised in another column. The writers miss the points that the Boat-Race of 1896 was one prolonged and continuous overstrain for the Cambridge crew, at any rate, from start to finish, and that the effects of overstrain of this kind does not make itself felt very often until years afterwards. The subject has many sides, is full of interest to athletes and practitioners, and we invite further correspondence.

### "SIXTEEN NERVOUS AND CARDIAC MAELSTROMS."

By Gilbert C. Bourne, New College, Oxford.

It would seem that the author of the article in The Hospital—himself, as his writing shows, of an excitable temperament—has been led, by the contemplation of the exertions of the sixteen men engaged in last Saturday's contest, to imagine sixteen nervous and cardiac maelstroms rending the frames of the unfortunate sixteen with results so dreadful that they can only be hinted at, not spoken. Intense nervous exhaustion, extreme cardiac strain, are the great factors for evil in this prolonged contest; so says one writer, with all the air of scientific authority.

Though I am not a member of the medical profession, nor yet a professed physiologist, I know something of physiology. I have taken part in two University races, and in a host of aquatic contests besides; I have trained more than one Oxford crew, and have been closely associated with the crews of the last thirteen years, and I beg leave to say that this picture of nervous excitement, violent cardiac tension, and all the rest of it, is absolute nonsense. An untrained man, rowing for a high wager, at high tension, over a short course, might possibly develop the alarming physiological symptoms imagined by the writer of the article. A trained University oar does not.

It is true that the effort required to row a hard race from Putney to Mortlake demands much from the heart and circulatory system in general. It is true that so much muscular exertion as is called forth involves the expenditure of much nervous energy, let alone the excitement of the contest. A man of ordinary physique would not be able to stand the strain, and a man of extraordinary physique would not be able to stand it if he were not carefully prepared for it. The men who row in the boat-race are men of extraordinary physique, and they are carefully prepared, under medical advice. The sixteen men who rowed last Saturday are the pick of some six thousand young men; they were submitted to a rigorous medical examination before practice began, and they were trained by experts for more than ten weeks before the race. Their diet, the times of their meals, the hours of their rising and going to bed, and their daily exercise have been the subject of the most careful thought and consideration, and they came to the post in a physiological state which very few doctors have any conception of. By a graduated and carefully planned scale of exercise, their hearts and lungs and muscular systems have been strengthened up to the point of standing the strain which was to be put upon them. Constant exercise in the boat has made the movements and even the exertions of rowing so much of a habit as to be almost mechanical: what would have been an intense effort to other men, involving great nervous waste and great cardiac strain, was to them part of a daily routine.

Within five minutes of the end of the race I was amongst the crews, and rubbed down several of the men. There was not a single case of exhaustion, the men scarcely seemed to be fatigued. I have seen most of them for two or three days after the race, and they were still in exceptionally good health. Yet our *à priori* physiologist assures us that "in every race every man cannot fail to be more or less a sufferer for a longer or shorter time." The fact is, sir, that the article in question is not founded, as every article pretending to scientific authority should be, on observed instances, but upon imagination. It is stated that the strain of rowing at high pressure for twenty minutes continuously is more than any human organism can bear without harm. There are scores of men who have done it, and done it many times, and I can assure you, sir, that the medical profession would soon starve if all men were so little dependent on their doctors as they are. A more flagrant, because a more definite, misstatement is this, "that it is certain that no race is ever run (? rowed) without serious injury to some." Has the writer of the article ever heard of or read Dr. J. E. Morgan's book on University oarsmen, published in 1873? It would effectually dispel his allusions as regards University oarsmen prior to that date; as regards oarsmen of a later date, it is my privilege to know most of them; many of them are my most intimate friends, and I can only say here that a more healthy and vigorous set of men I have never met anywhere.

It would take too much of your space if I were to attempt to give instances and discuss individual cases. I must conclude with this remark. The writer in The Hospital gives to his essay the grandiloquent title of "The Physiological Cost of the Boat-Race." Cost must be measured somehow; when I come to measure it up in my own case, and in those of my friends, I find it to include increased chest measurement, greater lung capacity, improved arterial tone, increased muscular development, a stronger heart. There is nothing that I can find to write down on the debit side of the account. The game, says our author, is not worth the candle; but if the candle has increased instead of diminishing during the progress of the game, it may, I think, be conceded that it is worth it.

---

### RACE ROWING IS NOT INJURIOUS TO HEALTH.

By G. Bridges Lewis, Broadstone.

The Hospital says race-rowing is injurious to health. Will you admit evidence on the other side? The famous Oxford crew who rowed with seven oars at Henley, and soon after, in full crew, at Putney in 1843, had two very severe races. Yet on the Jubilee in 1893 they were, all but two, alive and well. Of the two then departed, neither died, I believe, from the effects of rowing. One had died at the Cape, the other from congestion of the lungs. So much for the charge of physical evil results which anxious mothers dread; thoughtful fathers may also be free from any fear of moral evil results—as all these men have been steady, useful men in life.

I should like to know what the Senior Physician of St. George's says. Dr. Wadham had the glorious joy of rowing a very nearly dead-heat at Henley, and another hard race at Putney against our Oxford University Boat Club Four, n the summer of 1845. Some men are injured, no doubt, who are not fit—as some horses break down in training. There is common sense, plus medical advice, to be observed. An old schoolfellow of mine was a well-known and successful stroke, who was suddenly warned off from rowing in 1845; but he is alive now, and past 70—the father of 16 children. Do not let The Hospital frighten us from this splendid sport, which, more than any other, makes men of men.

